# A comparison between urban livestock production strategies in Burkina Faso, Mali and Nigeria in West Africa

**DOI:** 10.1007/s11250-012-0118-0

**Published:** 2012-03-20

**Authors:** Hamadoun Amadou, Luc Hippolyte Dossa, Désiré Jean-Pascal Lompo, Aisha Abdulkadir, Eva Schlecht

**Affiliations:** 1 Animal Husbandry in the Tropics and Subtropics, University of Kassel and Georg-August-Universität Göttingen, Steinstr. 19, 37213 Witzenhausen, Germany; 2 Organic Plant Production and Agroecosystems Research in the Tropics and Subtropics, University of Kassel, Steinstr. 19, 37213 Witzenhausen, Germany; 3 Plant Production Systems Group, Wageningen University, P.O. Box 430, 6700 AK Wageningen, The Netherlands

**Keywords:** Cattle, Chicken, Feeding, Health care, Small ruminants, Urban households

## Abstract

We undertook a comparative analysis of (peri-)urban livestock production strategies across three West African cities. Using a semi-structured questionnaire, livestock-keeping households (HH) were interviewed in Kano/Nigeria (84 HH), Bobo Dioulasso/Burkina Faso (63 HH) and Sikasso/Mali (63 HH). Questions covered livestock species kept, herd sizes and structure, feeds used, manure management, livestock marketing and production constraints. Sheep and goats dominated (*p* < 0.001) in Kano (76 and 75 % of HH) compared to Bobo Dioulasso (48 and 40 %) and Sikasso (28 and 40 %), while cattle and poultry were more frequent (*p* < 0.001) in Bobo Dioulasso (82 and 69 % of HH) and Sikasso (65 and 79 %) than in Kano (29 and 20 %). Across cities, ruminant feeding relied on grazing and homestead supplementation with fresh grasses, crop residues, cereal brans and cottonseed cake; cereal grains and brans were major ingredients of poultry feeds. Cattle and sheep fetched highest prices in Kano, unit prices for goats and chicken were highest in Sikasso. Across cities there was little association of gardens and livestock, whereas field cropping and livestock were integrated. There was no relation between the education of the HH head and the adoption of improved management practices (*p* > 0.05), but the proportion of HH heads with a long-term experience in UPA activities was higher in Kano and in Bobo Dioulasso than in Sikasso (*p* < 0.001). We therefore postulate that the high illiteracy rate among (peri-)urban livestock keepers in West Africa does not threaten the acceptance of improved technologies and innovations supporting the sustainability of their livestock production.

## Introduction

The mainstay of the economy of most West African countries is agriculture. The contribution of the livestock sector to the agricultural gross domestic product is 49, 15 and 44 % for Nigeria, Burkina Faso and Mali, respectively. While the growth rate of the regional animal production is estimated at 4 % per year, the regional demand for livestock products is expected to increase by >250 % until 2025 as compared to 2005 (SWAC-OECD/ECOWAS 2008). Given the current degree of urbanization of about 20 % and an annual growth rate of the urban population of 5–7 %, an important proportion of the total population of sub-Saharan West Africa will live in cities by the year 2050 (FAO 2011). Agricultural intensification will take place mainly in areas with good infrastructure and well-developed input and commodity markets, such as in and around urban centers (Graefe et al. 2008). Urban agriculture is a widespread practice in West Africa (Kutiwa et al. 2010) but has been neglected for a long time by policy makers. However, during the last two decades, the interest in food production in and around West African cities has increased together with the fast-growing urban population. The contribution of urban and peri-urban agriculture (UPA) to food security, household income and job creation has then been well recognized, along with the environmental benefits and problems resulting from its practices (Cohen and Garrett 2010; De Zeeuw et al. 2011). Livestock keeping constitutes an important part of UPA, but has received less attention than crop and vegetable cultivation. As a consequence, information on its extents, problems and potentialities is limited. The types of animals kept include cattle for milk and meat, small ruminants for meat, and poultry for eggs and meat. Previous study in Niamey, Niger (Graefe et al. 2008), reported that more than half of the households involved in UPA were rearing livestock. In Kano, Nigeria, Muhammad (2008) found that keeping livestock was a considerable source of additional income for civil servants and traders. Generally, each of these case studies emphasized the growing numbers of urban households involved in livestock rearing, and identified related constraints and opportunities. However the conclusions drawn and the recommendations made are not necessarily valid for the whole West African region because cities differ in size, structure, degree of urbanization, development history, environment, socioeconomic and cultural circumstances (Cissé et al. 2005). Furthermore, the legal framework within which urban livestock husbandry in particular is practiced, varies across West African countries and cities (Cissé et al. 2005) and may significantly affect locally observed management practices, production strategies and levels of crop-livestock integration. Cross-country comparisons are therefore needed to confirm if the findings of scattered case studies are to be developed into region-wide recommendations towards sustainable urban and peri-urban livestock production systems. Based on the above past observations, this study was carried out to compare the management strategies applied by livestock producers in three West African cities.

## Material and methods

### Study locations

The study was carried out in the three secondary West African cities of Kano, Nigeria (12°00 N, 8°31 E, 476 m a.s.l.), Bobo Dioulasso, Burkina Faso (11°16 N, 4°31 W, 460 m a.s.l.) and Sikasso, Mali (11°19 N, 5° 40 W, 410 m a.s.l.).

Kano is the second largest city of Nigeria and is located in the Sudano–Sahelian zone. It is currently populated by approximately 3.4 million people (UNPD 2009). Its climate is characterized by a dry season (October–May) and a rainy season (June–September). The annual average rainfall is about 700 mm (Kasali 2011).

Bobo Dioulasso is the second largest city of Burkina Faso, situated in the southern Sudanian savanna. It covers an area of 13,678 ha and hosted about 489,967 inhabitants in 2007 (Zida-Bangre 2009). Its climate is characterized by a rainy season (May–October), a cool dry season (November–February) and a hot dry season (March–May). The average annual rainfall varies between 900 and 1,200 mm.

Sikasso is situated in the southern part of Mali, in the southern Sudanian savanna. Hosting about 200,000 inhabitants, it covers an area of 3,745 ha (Ministère de l'Habitat et de l'Urbanisme 2005). The climate in Sikasso is similar to that of Bobo Dioulasso with an average annual rainfall of 800 to 1,100 mm.

### Data collection

A baseline survey using a semi-structured questionnaire was conducted simultaneously in the three cities between March and June 2007. A total of 335 (peri-)urban farm households (99 in Kano, 111 in Bobo Dioulasso and 125 in Sikasso) were selected and interviewed following a snowball sampling approach. The difference in sample size was due to the incomplete information gathered in some households in Kano and Bobo Dioulasso, which were removed from the data set. The questionnaire included questions related to the households’ socio-demographic (household size, age and sex structure, main occupation and formal education of its members) and economic characteristics, and the characteristics of their gardening, livestock and field cropping activities. The attendance (yes or no) of any formal school (primary, secondary or university) by the household head was used as proxy for his/her formal education. Data on livestock production were collected and included species kept; herd size, structure and dynamics; feeds and feeding strategies; manure management; health and diseases problems; access to veterinary services and uses of veterinary products, as well as livestock marketing. The households were classified into six farm types (Dossa et al. 2011). In the current study, only households that were keeping livestock were considered; they were classified into three farm types which are specified in Table 1, together with their spatial distribution.

**Table 1:** Distribution of the interviewed livestock keepers (*n*) across farming systems and across the three West African cities of Kano (Nigeria), Bobo Dioulasso (Burkina Faso) and Sikasso (Mali)

Production system	Total	Kano	Bobo Dioulasso	Sikasso
Commercial gardening plus field crops and livestock (cGCL)	88	58	21	9
Commercial gardening plus semi-commercial livestock (cGscL)	13	13	0	0
Commercial livestock plus subsistence field cropping (cLsC)	109	13	42	54

For classification and description of farming systems see Dossa et al. (2011)

### Data analysis

Data analysis was done with SPSS/PASW version 18.0 (SPSS Inc. 2010). The family labor in man-day equivalents was calculated according to the definition of the International Labor Organization of working age group which excludes household members below 15 years, and by applying conversion factors to male and female household members in different age groups as follows: 1.0 for males aged between 16 and 55 years, 0.75 for females between 16 and 55 years, 0.75 for males above 55 years and 0.5 for females above 55 years. Only non-household members who received a salary from the household against the performance of any kind of tasks in the livestock unit were considered as hired laborers.

Descriptive statistics were performed for all variables. Differences between groups within and across cities were explored using analysis of variance (ANOVA) and least significant difference (LSD) post-hoc comparison for normally distributed continuous variables. For not normally distributed variables, the Chi-square test (categorical variables) and the Kruskal–Wallis test followed by the Mann–Whitney *U* test for post hoc separation of group means (continuous variables) were used with significance declared at *p* < 0.05.

Logistic regression was used to assess the odds of livestock keepers’ adoption of supplementary feeding of livestock (yes/no), the use of veterinary services (yes/no), curative medical treatment (yes/no) and prophylactic vaccination (yes/no) of their animals from a set of independent predictor variables. The latter included city (location), socio-economic characteristics such as formal education, geographical origin (native of city/migrant) and years of experience in urban agriculture of head of household, practice of gardening and field cropping, number of livestock species kept and total number of tropical livestock units (TLU^1^) owned. We performed a stepwise logistic regression procedure with backward elimination of predictors (Hair et al. 2009), whereby the analysis began with the full model (Eq. 1) that included all predictor variables. Variables that were not useful in predicting the dependent variables were eliminated automatically from the model in an iterative way.

1 $$ {\text{Logit}}\;(Y) = \alpha + {\beta_1}{X_1} + {\beta_2}{X_2} + \cdot \cdot \cdot + {\beta_n}{X_n} $$

Where *Y* is the dependent variable*, α* the *Y* intercept, *β* the regression coefficient and *X* the predictor.

The positive or negative sign of the coefficient *β* indicates the direction of the relationship between a given independent variable and the dependent variable, while the odds ratio (*e* ^*β*^) indicates the magnitude of change in the probability of the dependent variable event in case of a one unit change in the independent variable. The fit of the final model was assessed by the model Chi-square (model *χ*²) and the goodness-of-fit test of Hosmer and Lemeshow (Archer and Lemeshow 2006). Well-fitting models showed significance (*p* ≤ 0.05) on the model *χ*² and non-significance (*p* > 0.05) on the goodness-of-fit test of Hosmer and Lemeshow.

## Results

### Household socio-economic characteristics 

Out of the 335 households (HH) surveyed, 210 were involved in animal husbandry. In Kano and Sikasso, a higher proportion of livestock keeping HH was involved in gardening activities (Table 2) compared to Bobo Dioulasso (*χ* ^2^ = 42.7, *p* < 0.001), whereas in Bobo Dioulasso livestock keeping was mainly combined with field cropping. In Kano and Sikasso, the head of a livestock-keeping HH was more frequently native of the city than in Bobo Dioulasso, while a higher proportion of HH heads had long-term experience in UPA activities in Kano and Bobo Dioulasso than in Sikasso (*χ* ^2^ = 42.2, *p* < 0.001). Livestock-keeping families mainly belonged to the Hausa, Bobo-Dioula and Sénoufo ethnic groups, which are the dominant groups in Kano, Bobo Dioulasso and Sikasso, respectively. In Kano, a lower proportion of HH heads (16 %) had formal education compared to Bobo Dioulasso and Sikasso (*χ* ^2^ = 14.5, *p* < 0.01).

**Table 2:** Key characteristics of livestock keeping households (HH) across the three West African cities of Kano (Nigeria), Bobo Dioulasso (Burkina Faso) and Sikasso (Mali)

Variables	Kano (*n* = 84)	Bobo Dioulasso (*n* = 63)	Sikasso (*n* = 63)	*χ* ^2^	*p*≤
Formal education of HH head^a^				14.5	0.001
No (%)	79	69	49		
Yes (%)	21	31	51		
Origin of HH head^a^				42.2	0.001
Native (%)	92	43	74		
Immigrant (%)	7	57	26		
Gardening ^a`^				42.7	0.001
Yes (%)	82	31	70		
No (%)	18	69	30		
Field crop cultivation				1.7	n.s.
Yes (%)	92	97	92		
No (%)	8	3	8		
Experience in urban agriculture^a^				40.3	0.001
<5 years (%)	4	11	31		
6–10 years (%)	5	14	25		
>10 years (%)	91	75	44		
					
		Mean ± SD			
Age of HH head (years)	48.1 ± 12.81	51.6 ± 14.87	50.0 ± 15.71		n.s.
HH members formal education (*n*)	4.5 ± 4.34	5.7 ± 3.13	6.6 ± 8.55		n.s.
Family labor (*n*)	8.3^b^ ± 7.03	7.6^b^ ± 2.01	12.2^c^ ± 11.14		0.05
Hired labor (*n*)	0.5^b^ ± 1.10	0	1.6^c^ ± 3.18		0.05

*n.s.* not significant

^a^Upper part of table: significant differences between cities; Chi-square test

^b,c^Lower part of table: significant differences between means with different superscripts; Kruskal–Wallis test.

Regardless of city, the major livestock species kept included cattle, small ruminants, donkeys and chicken (Table 3). The proportion of HH keeping cattle was higher in Bobo Dioulasso than in Sikasso and Kano (*χ* ^2^ = 45.7, *p* < 0.001), whereas the proportion of HH keeping small ruminants was highest in Kano (*χ* ^2^ = 33.6, *p* < 0.001).

**Table 3:** Species kept and herd sizes in livestock keeping households (HH) across the three West African cities of Kano, Nigeria (84 HH), Bobo Dioulasso, Burkina Faso (63 HH) and Sikasso, Mali (63 HH)

Variable	Kano		Bobo Dioulasso		Sikasso		*χ*²	*p* ≤ ^a^
HH keeping:	*n*	%	*n*	%	*n*	%		
Cattle	24	29	52	82	41	65	45.7	0.001
Small ruminants	75	95	39	60	36	59	33.6	0.001
Donkeys	6	7	29	46	31	49	38.5	0.001
Chicken	17	20	44	69	50	79	60.9	0.001
								
	Mean	SD	Mean	SD	Mean	SD		*p* ≤ ^b^
Species kept (*n*)	2.1^b^	1.04	2.9^a^	1.33	2.6^a^	1.14		0.001
Cattle (TLU)^c^	22.0	29.94	18.5	28.93	15.0	18.16		n.s.
Small ruminants (TLU)^c^	1.7	1.67	1.2	1.10	1.4	1.31		n.s.
Donkeys (TLU)^c^	1.7^a^	1.04	0.6^c^	0.29	0.8^b^	0.45		0.05
Chicken (TLU)^c^	0.2^a^	0.26	0.4^ab^	0.69	3.7^b^	11.76		0.05
Total livestock (TLU)^c^	8.2^a^	19.55	22.0^c^	27.59	13.9^bc^	20.91		0.05

*n.s*. not significant; *TLU* tropical livestock unit

^a^Upper part of table: significant differences between cities; Chi-square test

^b^Lower part of table: significant differences between means with different lowercase letters (a, b, c); Kruskal–Wallis test

^c^TLU, hypothetical animal of 250 kg live weight. Conversion factors used: cattle = 0.80, sheep and goats = 0.10, donkey = 0.5; chicken = 0.01

The average number of species kept per HH in Bobo Dioulasso and Sikasso was higher (*p* < 0.001) than in Kano. Farms of type commercial livestock plus subsistence field cropping (cLsC) kept a greater variety of species than cGCL farms. Within cLsC farms, the number of species kept was higher in Bobo Dioulasso than in the other two cities (*p* < 0.05). The average number of TLU per HH was higher (*p* < 0.01) in Bobo Dioulasso than in Sikasso and Kano. However, there was no significant difference between the HH of the three cities in the average size of cattle and small ruminant herds. Across cities, 54 % of the livestock keepers raised chicken, whereby the number of HH keeping backyard chicken was higher (*χ* ^2^ = 13.6, *p* < 0.01) than the number of commercial chicken keepers (broilers or layers). Between the three cities, no significant differences were determined for the average number of chicken in backyard holdings (18 ± 12.5 across cities) and average number of chicken on commercial farms (654 ± 1476.1 across cities), respectively.

### Livestock feeding strategies

In addition to year-round daily pasturing in open city spaces and on fallows and rangelands at the cities’ peripheries, supplement feeds were offered to ruminant livestock, whereby feeding strategies differed with type of animal and orientation of production, such as draught animals, dairy cows, beef cattle and small ruminants. Similarly, different feeding strategies were used for broilers and layers. Households keeping cattle, small ruminants and chicken, respectively, differed in the types of supplement feeds used.

**Fig. 1:**
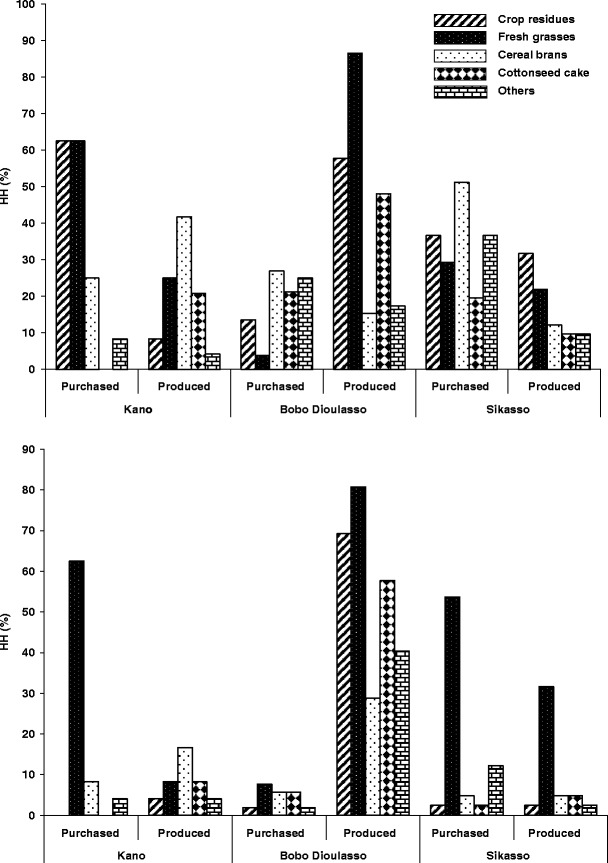
Number of cattle-keeping households offering different types of purchased or produced feed in the dry season (*above*) and rainy season (*below*) in the West African cities of *Kano* (Nigeria), *Bobo Dioulasso* (Burkina Faso) and *Sikasso* (Mali)

In Kano, 29 and 13 % of all cattle herds (*n* = 24) were purely relying on grazing in the rainy and the dry season, respectively. In the dry season, 10 and 32 % were purely relying on grazing in Bobo Dioulasso and Sikasso. During the rainy season, at least one type of supplement feed was given to 71, 88 and 88 % of the cattle herds in Kano, Bobo Dioulasso and Sikasso, while respective dry season values were 87, 90 and 68 %. Supplementation of cattle was mainly based on fresh grasses, crop residues, cereal brans and cottonseed cake (Fig. 1). The proportion of farmers who fed crop residues to their animals was slightly higher (*χ* ^2^ = 25.3, *p* < 0.001) in Bobo Dioulasso than in the two other cities. While significantly higher during the dry season (*χ* ^2^ = 62.7, *p* < 0.001), the proportion of farmers who fed fresh grasses to their cattle was significantly lower (*χ* ^2^ = 3.8, *p* > 0.05) in Kano than in Sikasso and Bobo Dioulasso during the rainy season. Fresh grasses were mostly purchased in Kano, but were self-produced by the farmers in Bobo Dioulasso. Feeding cottonseed cake and cotton grain to cattle was more commonly observed in Bobo Dioulasso than in Kano and Sikasso. Other types of cattle feed included bush hay, cowpea hay and groundnut hay, vegetable residues, brewery wastes and salt. Legume hays were offered by 20 and 40 % of cattle farmers in Bobo Dioulasso and Sikasso during the dry seasons, whereas brewery wastes were only used in Bobo Dioulasso. Few HH in Bobo Dioulasso and in Sikasso offered vitamins to their cattle.

**Fig. 2:**
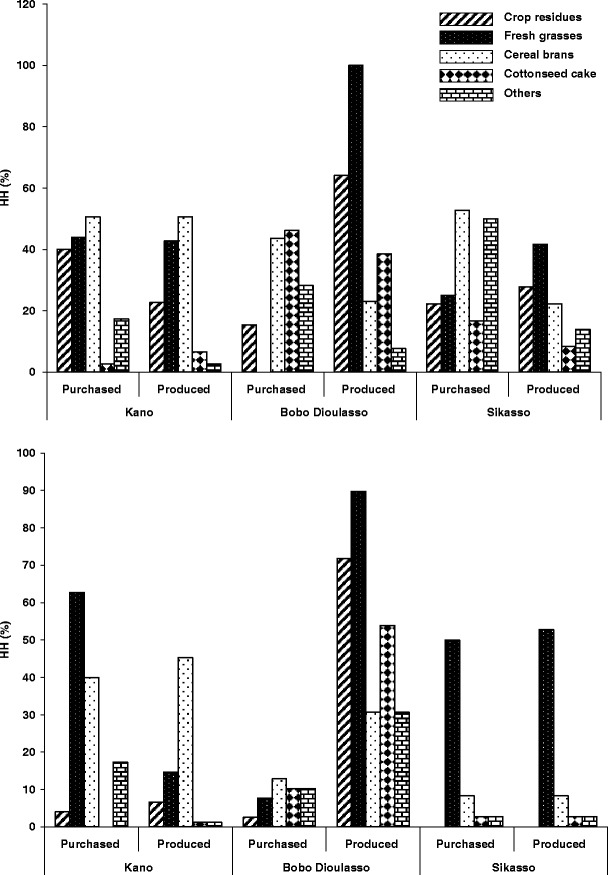
Number of small ruminant keeping households offering different types of purchased or self-produced feed in the dry season (*above*) and rainy season (*below*) in the West African cities of *Kano* (Nigeria), *Bobo Dioulasso* (Burkina Faso) and *Sikasso* (Mali)

At least one type of supplement feed was offered to 95, 100 and 72 % of the small ruminant herds in Kano, Bobo Dioulasso and Sikasso in the dry season, while in the rainy season this proportion was >94 % in all cities. Similarly to cattle, fresh grasses, crop residues and cottonseed cake were the main feeds for small ruminants (Fig. 2). Purchased cottonseed cake was mainly distributed in Bobo Dioulasso, while brans from maize, millet, sorghum and rice were used by a higher proportion of HH in Kano than in Bobo Dioulasso and Sikasso (*χ* ^2^ = 8.7, *p* < 0.05). During the rainy season, fewer HH in Bobo Dioulasso (*χ* ^2^ = 78.4, *p* < 0.001) than in Kano and Sikasso offered purchased fresh grasses to their sheep and goats. Other types of feed distributed to small ruminants included purchased vegetable residues and self-produced hay in Kano, cotton seed grain in Bobo Dioulasso and leguminous leaves in Sikasso. Few HH regularly offered salt to their small ruminants.

There was a remarkable preference for self-compounded feeds among poultry farmers in all cities; the use of commercial feed mash was uncommon and was only observed among a few egg and broiler producers in Sikasso and Kano. With insignificant differences between cities and seasons, respectively, cereal grains and brans were the main ingredients of poultry feeds, whereby the proportion of HH purchasing cereal brans was higher in Bobo Dioulasso than in Kano and Sikasso (*χ* ^2^ = 17.9, *p* < 0.05). At the same time, a higher proportion of HH in Bobo Dioulasso than in Kano and Sikasso (*χ* ^2^ = 17.9, *p* < 0.05) also produced cereal grains offered to poultry. Additional types of chicken feed included fresh grasses in Bobo Dioulasso and Sikasso, cottonseed cake in Bobo Dioulasso, commercial feed mash in Kano and home-made feed mash in Sikasso. Twenty percent of the poultry units in Sikasso could be classified as semi-commercial broiler and layer farms which regularly offered minerals and vitamins.

The results of the logistic regression (Table 4) indicated that livestock keepers’ decision to offer supplementary feed was significantly affected by the city and the animal species kept. Cattle were more likely to receive supplement feed (*e* ^*β*^ = 4.6) than small ruminants and chicken, and supplementation was more likely to be practiced in Bobo Dioulasso (*e* ^*β*^ = 5.9) than in the two other cities.

**Table 4:** Parameters of the logistic regression analysis for variables predicting the adoption of supplementary feeding, use of veterinary services, vaccination and curative medical treatments across 210 urban and peri-urban farm households (HH) in the West African cities of Kano, Nigeria (84 HH), Bobo Dioulasso, Burkina Faso (63 HH) and Sikasso, Mali (63 HH)

Predictors	Parameters					
	*β*	SE *β*	Wald’s *χ* ^2^	*df*	*p*≤	Odds ratio
**Adoption of supplementary feeding**						
Constant	−3.242	1.054	9.452	1	0.002	n.a.
Site			6.278	2	0.043	
Site 1 (Kano)	0.087	1.069	0.007	1	0.935	1.091
Site 2 (Bobo Dioulasso)	1.787	0.885	4.709	1	0.043	5.970
Species 1 (cattle)	1.527	0.704	4.709	1	0.030	4.603
Species 2 (small ruminant)	−1.072	0.704	2.322	1	0.128	0.342
Species 3 (chicken)	−0.672	0.681	0.976	1	0.323	0.510
Overall model evaluation (model *χ* ^2^)			15.858	5	0.007	
Goodness-of-fit test (Homer and Lemeshow)			10.222	7	0.176	
**Use of veterinary services**						
Constant	−4.216	0.746	31.976	1	0.000	n.a.
Site			17.140	2	0.000	
Site 1 (Kano)	1.164	0.669	3.021	1	0.082	3.201
Site 2 (Bobo Dioulasso)	2.937	0.747	15.444	1	0.000	18.854
Species 1 (cattle)	2.117	0.555	14.577	1	0.000	8.310
Overall model evaluation (model *χ* ^2^)			34.925	3	0.000	
Goodness-of-fit test (Hosmer and Lemeshow)			5.577	4	0.233	
**Curative medical treatment**						
Constant	−1.735	0.362	23.018	1	0.000	n.a.
Site			46.039	2	0.000	
Site 1 (Kano)	−2.440	1.071	5.194	1	0.023	0.087
Site 2 (Bobo Dioulasso)	2.833	0.502	31.862	1	0.000	17.000
Overall model evaluation (model *χ* ^2^)			81.710	2	0.000	
Goodness-of-fit test (Hosmer and Lemeshow)			0.000	1	1.000	
**Prophylactic vaccinations**						
Constant	0.067	0.258	0.067	1	0.796	n.a.
Site			35.146	2	0.000	
Site 1 (Kano)	4.108	1.040	15.593	1	0.000	60.806
Site 2 (Bobo Dioulasso)	−2.121	0.541	15.384	1	0.000	0.120
Overall model evaluation (model *χ* ^2^)			104.979	2	0.000	
Goodness-of-fit test (Hosmer and Lemeshow)			0.000	1	1.000	

For explanation of parameters see “Data analysis” Section

*n.a.* not applicable

### Healthcare practices 

Overall, and regardless of livestock species kept, 85 % of all livestock-keeping HH stated to provide prophylactic vaccination or treatments to their animals. Across cities, the majority (68 %) of HH keeping more than one animal species only provided health care to species that they considered of economic importance and wherever cattle were kept these were given priority over all other species.

With no significant differences between farm types, the large majority of livestock keepers in Sikasso (95 %), Kano (78 %) and Bobo Dioulasso (70 %) acknowledged to have access to veterinary services. Prophylactic vaccinations of cattle and small ruminants were provided by a significantly higher (*χ* ^2^ = 41.9, *p* < 0.001) proportion of HH in Sikasso (85 %) and Kano (98 %) than in Bobo Dioulasso (25 %). In contrast, a significantly higher (*χ* ^2^ = 42.2, *p* < 0.001) proportion of HH in Bobo Dioulasso (89 %) than in Sikasso (48 %) and Kano (2 %) reported to provide curative treatments to their ruminant animals (Table 4). The semi-commercial poultry keepers particularly in Sikasso (18 %) were observing a time table for prophylactic vaccinations against the major poultry diseases such as Newcastle disease, infectious bursal disease and infectious bronchitis, including also treatments against internal and external parasites. Almost all livestock keepers had their animals confined to closed barns or sheds during the night in the courtyard or in the vicinity of their houses.

Similar to the practice of feed supplementation, the results of the logistic regression (Table 4) showed that city and animal species were the most important factors determining the use of veterinary services. The likelihood of using veterinary services was much greater in Bobo Dioulasso (*e* ^*β*^ = 18.8) than in Kano and Sikasso. City was the single factor that significantly predicted the use of curative medical treatments and prophylactic vaccinations. Regardless of the species kept, livestock keepers in Bobo Dioulasso were more likely (*e* ^*β*^ = 17.0) to provide curative medication to their animals than those in Kano and Sikasso. In contrast, livestock keepers in Kano were more likely (*e* ^*β*^ = 60.8) than those in Bobo Dioulasso and Sikasso to prevent occurrence of diseases in their herd through prophylactic vaccinations.

### Manure production and uses

Livestock faeces and urine, sometimes mixed with bedding material and feed leftovers, were commonly heaped without any cover in the courtyard close to the barn or around the house. From there, the dung was removed after a long period of exposure to high temperatures during the hot dry season and considerable rainfall during the rainy season. Across the three cities, livestock dung was either burnt together with other household wastes (30 % of HH) or used as fertilizer in crop fields and/or gardens (39 % of HH). Burning occurred mainly in Kano and was reported by 71 % of HH that kept only a few heads of small ruminants. In contrast to Kano, the large majority of HH in Bobo Dioulasso (97 %) and Sikasso (95 %) used manure as fertilizer. In Bobo Dioulasso, manure was only used on crop fields, whereas in Sikasso it was applied to crop fields and vegetable gardens. Only a few HH in Sikasso (5 %) did sell manure. There were also differences between farm types with respect to manure uses: Manure was burnt in almost half of the cGCL farms, whereas in 85 % of the cLsC farms, it was applied to crop fields or gardens. For the latter farm type, manure use as fertilizer was more frequent in Sikasso (94 %) and in Bobo Dioulasso (95 %) than in Kano (15 %).

### Marketing of livestock

**Fig. 3:**
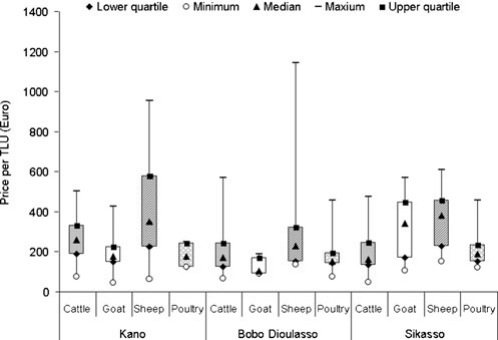
Average (2006–2007; *n* = 279) sales prices of different livestock species in the West African cities of *Kano* (Nigeria), *Bobo Dioulasso* (Burkina Faso) and *Sikasso* (Mali)

Overall, sales were the main reason for animal offtakes, and a significantly higher proportion of animals was sold (70 %) than purchased (39 %) by the livestock keepers involved in this study. Purchases were more common in Sikasso than in the other two cities, whereas sales were more frequent in Kano and Bobo Dioulasso than in Sikasso. Usually both types of livestock transactions were taking place in the dry season. Cattle and sheep fetched higher prices in Kano (211 € ± 84.5 and 42 € ± 24.2, respectively) than in Bobo Dioulasso (153 € ± 81.2 and 31.5 € ± 30.1, respectively) and in Sikasso (160 € ± 73.3 and 36 € ± 14.4, respectively). In contrast, prices for one goat and one unit of poultry were not significantly different between Sikasso (32.80 € and 2.00 €, respectively), Kano (19.20 € and 1.80 €, respectively) and Bobo Dioulasso (12.20 € and 1.80 €, respectively). The TLU unit price of sheep was higher than that of the other animal species (Fig. 3).

## Discussion

### Households' socio-economic characteristics

The high proportion of HH (63 %) that were keeping livestock across the three cities highlighted that livestock production was a popular activity of urban dwellers in the surveyed locations. This proportion was lower than the 82 % observed in Niamey where a systematic sampling of urban households had been adopted (Graefe et al. 2008). The relatively young age of the large majority of HH heads across cities compared well to earlier findings from Kano (random sampling of households; Muhammad 2008) and Niamey (systematic sampling of households; Belli et al. 2008), and indicates that livestock keeping will continue to flourish in West African cities in the near future. Livestock-keeping HH in Kano and Bobo Dioulasso had more years of experience in UPA than their counterparts in Sikasso. Muhammad (2008) indicated that this factor can be determinant for the management of (peri-)urban livestock; however, our logistic regressions indicated that there was no impact of years of experience on innovation uptake.

Most of the surveyed HH kept more than one livestock species, which is in agreement with a previous study in Niamey (Graefe et al. 2008). Akter et al. (2007) argued that the commonly observed species diversification is a risk-reduction strategy.

Higher annual rainfall and lower population density in Bobo Dioulasso and Sikasso as compared to Kano implied a higher availability of feed for animals. This was probably the reason for the lower total number of ruminants and lower proportion of households keeping cattle in Kano compared to Bobo Dioulasso and Sikasso (Muhammad 2008). With respect to the average size of cattle herds, no significant differences were observed between cities. However, the values obtained in our study were higher than the average of 1.4–2.4 TLU of cattle reported by Diogo et al. (2010) for Niamey and lower than numbers reported by Mattoni et al. (2007) for dairy herds in peri-urban Bobo Dioulasso.

Similar to cattle, the average size of small ruminant herds in our study was higher than the eight heads per HH reported by Rischkowsky et al. (2006) for Bobo Dioulasso, but comparable to the values reported by Diogo et al. (2010) for Niamey. In Kano, cLsC farms possessed more TLU than their counterparts in Bobo Dioulasso and Sikasso. They were mainly involved in buying, fattening and selling small ruminants, and some of them also practiced intensive poultry farming. Hamadou et al. (2008) reported that small ruminants were commonly kept in and around West African cities, mainly for generating cash income, and for ceremonies and other social purposes.

### Livestock feeding 

Regardless of the city, small ruminants were mostly zero-grazed and all HH provided their cattle with additional feeds to complement the lack of grazing area in open inner-city spaces as well as on fallows and rangelands located at the outskirts of the towns. These findings were consistent with those of Hamadou et al. (2008) and Duku et al. (2010) who indicated that most (peri-)urban livestock keepers respond to declining grazing areas by shifting from the traditionally extensive towards more intensive feeding practices. Land is the fundamental resource required for farming, and access and tenure are critical (IFAD 2008). Since more than 90 % of livestock-keeping HH in all three cities were also involved in field cropping, crop residues were mostly produced on the farms. The more pronounced use of cottonseed cake in Bobo Dioulasso and Sikasso was mainly due to the presence of cotton-processing factories in these cities that are located in West Africa’s cotton zone. Generally, farmers supplemented their animals to allow them express their productive performance in terms of weight gain, milk yield and ability to reproduce (Sidibé-Anago et al. 2008; Mapato et al. 2010). The difference between Bobo Dioulasso and the other cities in supplementation practices can partly be explained by the dissemination of some of these technologies by a dairy development project in the 1990s (Hamadou et al. 2008). However, in a recent study of (peri-)urban livestock production in Niamey, Diogo et al. (2010) observed that the quantity of feed and nutrients offered to animals by far exceeded their requirements and finally ended up at the dung heap in form of faeces, urine and feed refusals. The high costs of commercial poultry feeds and negative experiences of poultry farmers with respect to the quality of these feeds (Apantaku et al. 2006) were probably the main reasons for the preference of self-compounded feeds.

### Healthcare practices 

The high proportion of HH that reported to vaccinate or medically treat their animals points to an increased importance of healthcare among urban livestock keepers (Hamadou et al. 2008). However, few HH took care of all animals, reflecting a species prioritization. This prioritization was probably associated with production objectives: small ruminants were given high priority in Kano because they were imperative for religious celebrations (Ajala et al. 2008). As indicated by the logistic regression, livestock keepers in Bobo Dioulasso and Sikasso were less likely than those in Kano to vaccinate their animals against infectious diseases. Consequently, one might expect a higher occurrence of infectious diseases among livestock herds in these two cities, and the higher likelihood of using a curative medical treatment in Bobo Dioulasso compared to Kano indicated that zoonotic as well as other infectious diseases were more prevalent in Bobo Dioulasso. Thys et al. (2005) reported that the first constraint of animal husbandry in Ouagadougou was animal health including disease occurrence and drug and treatment delivery. Differences in the adoption of curative medical treatments between cities might also be related to cultural differences as well as differences in types of livestock production systems, environmental factors and husbandry methods (Heffernan et al. 2011). Pollock et al. (2011) emphasized the need for collaboration between public health sectors (veterinary and human) and all other services involved in urban policy planning and management to achieve satisfactory urban hygiene.

### Manure utilization 

Manure production was influenced mainly by the objectives of livestock production, herd size and management strategies (Powell et al. 2008). The frequent use of animal dung for vegetable gardens and crop fields in Bobo Dioulasso and Sikasso agreed with recent findings in Niamey (Graefe et al. 2008), reflecting an increasing awareness of (peri-)urban farmers of the importance of manure for maintaining soil fertility.

The majority of the HH in our study locations used to heap livestock dung in the courtyard. Unprotected dung storage potentially affects dung quality by providing favorable conditions for pathogens and parasites, and in consequence bears contamination risks for manured vegetables (Diogo et al. 2010). The exposure of the dung heap to high temperatures during the hot dry season and to rainfall during the rainy season also leads to nutrient losses through gaseous emissions, leaching and runoff (Predotova et al. 2010). There is thus need to raise farmers’ awareness of the importance of dung management in view of efficient nutrient recycling and minimized risks for human and environmental health.

### Livestock marketing

The higher proportion of animals sold than purchased by the livestock keepers involved in this study indicated that (peri-)urban livestock keeping was strongly market oriented. Cohen and Garrett (2010) reported that with the food crisis in 2007–2008 the vulnerability of poor urban dwellers increased and food purchases accounted for the bulk of their expenditures. Tefera (2010) stated that in Central Ethiopia, urban agriculture plays an important role in attaining household food security. He argued that households owning a higher quantity and quality of livestock are less likely affected by food insecurity. The higher prices fetched by cattle and sheep in Kano was probably due to the city’s high degree of urbanization as well as high population density; moreover, the majority of Kano’s population is Muslim and request for sheep was high with religious ceremonies such as Eid al Kabir. However, across the region the livestock sector still receives little support in the form of public investment in processing and packaging infrastructure and lacks policies that stimulate regional trade in animal products (SWAC-OECD/ECOWAS 2008).

### Implications for the design of programs to improve management practices and animal performances

From northern Namibia, Musaba (2010) reported that high school education was positively related to the adoption of new cattle management technologies. Our study in contrast showed that the education level and the experience of HH heads in (peri-)urban agriculture did not affect the decision on adopting improved livestock management, such as supplementation and vaccination. This suggests that the high rate of illiteracy among livestock keepers in Kano, Bobo Dioulasso and Sikasso was not restricting the adoption of new technologies as suspected by Musaba (2010). Similar to our results, Onemolease and Alakpa (2009) found no relationship between level of education and adoption of improved livestock management practices such as animal vaccination, nutrition, deworming, isolation of sick animals and improved breeding practices in the Niger delta region of Nigeria.

Yet, our results suggest that the adoption of improved technologies is more or less pronounced depending on the livestock species, and higher adoption rates can be expected among cattle farmers. SWAC-OECD/ECOWAS (2008) reported the use of artificial insemination and crossbreeding by (peri-)urban dairy farmers to improve milk yield and body weight gain in cattle. In contrast to rural areas where cattle are still kept to accumulate wealth and prestige (Belli et al. 2008), cattle keeping in cities is mainly market oriented (Hamadou et al. 2008). Because grazing areas are shrinking due to urbanization, cattle farmers increasingly turn to semi-intensive production systems such as zero-grazing and supplementary feeding with concentrate feeds and better veterinary care. Small ruminants and local chicken have lower feed requirements and fulfill more non-commercial functions (Chukwuka et al. 2010) than cattle.

## Conclusions

Urban livestock keeping has a strong market orientation as evidenced by the relative higher number of animals sold than purchased by the livestock keepers in this study. The rapidly growing market demand will likely promote the intensification and modernization of the commercially valuable production branches (cattle, poultry). However, efficient resource use will determine economic profitability, environmental safety and risks to human health of these production systems. This will require wide dissemination of research results through government and private–public partnership and adoption by livestock keepers of improved feeding, healthcare and dung management. Our results suggest that this adoption will not be hindered by the illiteracy of livestock managers. However, in-depth market analysis is necessary for the improvement of the marketing and pricing systems.

## Footnotes

1 TLU, tropical livestock unit: standardized animal of 250 kg live weight; 1 cattle = 0.8 TLU; 1 donkey = 0.5 TLU; 1 sheep/goat = 0.1 TLU, 1 chicken = 0.01 TLU.
